# Influence of Alanine Transaminase Levels on Alpha-Fetoprotein for Predicting Hepatocellular Carcinoma in Patients with Hepatitis B Infection

**DOI:** 10.1155/2020/2043715

**Published:** 2020-12-15

**Authors:** Jiahao Chen, Manli Wu, Jiao Gong, Zhihuan Liu, Guowei He, Huiqing Zhu, Zhaoxia Li, Bo Hu

**Affiliations:** ^1^Department of Laboratory Medicine, Third Affiliated Hospital of Sun Yat-sen University, Guangzhou, China; ^2^Department of Ultrasound, Third Affiliated Hospital of Sun Yat-sen University, Guangzhou, China; ^3^School of Clinical Medicine, Guangdong Pharmaceutical University, Guangzhou, China

## Abstract

**Purpose:**

To investigate the influence of alanine transaminase (ALT) on the accuracy of alpha-fetoprotein (AFP) for detecting hepatocellular carcinoma (HCC).

**Methods:**

This retrospective study recruited 799 patients with HCC, cirrhosis, and chronic hepatitis due to hepatitis B infection and healthy adults between July 2017 and January 2019. Comparisons of the area under the receiver operating characteristic curves (AUCs) for detecting HCC in different ALT levels were calculated.

**Results:**

Serum ALT and gamma-glutamyl transferase levels were significantly associated with elevated AFP in patients without HCC. The AUC of AFP was higher in patients with ALT ≤ 2 upper limit of normal (ULN) than in patients with ALT > 2 ULN (0.806 vs. 0.611, *P* < 0.001). Nevertheless, there were no significant differences in the AUCs of AFP/(ALT × aspartate aminotransferase (AST)) in patients with ALT ≤ 2 ULN and with ALT > 2 ULN (0.745 vs. 0.769, *P* = 0.68). AFP/(ALT × AST) was better than AFP in patients with ALT > 2 ULN for detecting HCC (*P* < 0.001).

**Conclusions:**

Higher ALT levels might impair the accuracy of AFP for diagnosing HCC. AFP tests showed better accuracy in patients with ALT ≤ 2 ULN whereas the AFP/(ALT × AST) ratio was recommended in patients with elevated ALT levels.

## 1. Introduction

Hepatocellular carcinoma (HCC) is considered the fifth most common malignancy in the world with a high mortality rate [1], and it was estimated that half of the HCC cases and deaths occurred in China [2, 3]. The epidemiological observation suggests that hepatitis B viral infection is a potential risk factor for HCC [4, 5]. Therefore, cancer screening for the target population and early diagnosis of HCC is essential to improve life expectancy and reduce mortality.

Among serum tumor markers, alpha-fetoprotein (AFP) is the most widely used serological marker for HCC evaluation [6]. However, the accuracy of AFP for HCC detection has been intensely debated for relatively low accuracy [7–9]. Indeed, the updated American Association for the Study of Liver Diseases (AASLD) guidelines currently exclude AFP from surveillance testing and use ultrasonography as the optimal option for the detection of early HCC [10].

Numerous data have shown the nonspecific finding of significantly high serum AFP levels in patients with chronic liver disease and cirrhosis [6, 11]. There have been case reports with regard to abnormally elevated AFP levels in patients with chronic hepatitis B or C infections without evidence of HCC, and elevated serum AFP levels decreased in response to antiviral therapy [12–14]. Such results might explain that abnormal elevation of AFP levels can be due to hepatic inflammation and viral replication. However, data was scarce about what factors lead to an abnormal increase in AFP levels in patients with liver disease without developing HCC. Several studies have shown that the serum alanine transaminase (ALT) level was significantly associated with elevated AFP in patients without HCC [15–18]. A recent study illustrated that the diagnostic performance of the novel index AFP/[ALT × aspartate aminotransferase(AST)] was superior to that of AFP for HCC [19]. Hence, we hypothesize that increased ALT levels might impair the diagnostic accuracy of AFP for the detection of HCC.

Therefore, this retrospective study was aimed at exploring the influence of ALT levels on the diagnostic accuracy of AFP for the detection of HCC in comparison with the AFP/(ALT × AST) ratio.

## 2. Materials and Methods

### 2.1. Study Design and Patients

This was a retrospective study conducted at the Third Affiliated Hospital of Sun Yat-sen University (Guangzhou, China) between July 2017 and January 2019. Patients with liver disease due to hepatitis B viral infection (including the HCC group, group with cirrhosis, and group with chronic hepatitis B) and normal health adults (for the normal control group) were retrospectively recruited. The exclusion criteria of the study were as follows: (a) had a history of antiviral therapy at study entry as such therapy might have an impact on ALT and AFP levels and affect study results; (b) had a previous history of liver transplantation; (c) were younger than 18 years; (d) combined with other liver diseases (autoimmune liver disease, alcoholic liver disease, nonalcoholic fatty liver disease, etc.); (e) had a previous history of tumor treatment for HCC (surgery, ablation therapy, or chemoradiotherapy); and (f) unavailable AFP values.

The study protocol was approved by the Ethics Committee of the Third Affiliated Hospital of Sun Yat-sen University, Guangzhou, China. Informed written consent was obtained from all recruited patients.

### 2.2. Clinical Diagnostic Criteria

The HCC diagnosis in the study was confirmed according to the European Association for the Study of the Liver clinical practice guidelines [4]. Newly developed HCC cases were diagnosed by histological findings or at least one positive imaging technique (contrast-enhanced CT, contrast-enhanced MRI or CEUS) for nodules of ≥1 cm in diameter for high-risk individuals.

Chronic hepatitis B viral infection was diagnosed for the positive findings of the hepatitis B surface antigen for at least 6 months. Cirrhosis was diagnosed based on the clinical, biochemical, and ultrasonic findings or biopsy results, as described in previous studies. Normal healthy adults were confirmed without liver disease according to serological and ultrasonic results.

### 2.3. Measurements of AFP, ALT, and AST

The enrolled patients were required to fast overnight, and all serum samples were collected and measured in the morning. Serum levels of AFP were measured using electrochemiluminescence (Cobas E601, Roche, Inc., Germany) with an upper limit of detection of 1210 ng/mL. Serum levels of ALT and AST were measured using biochemical rate assay (Hitachi 7600, Japan). A normal ALT level was interpreted as ≤35 U/L.

All serum sample tests were performed by two experienced inspectors (J.C. and Z.L. with experience of >4 years in clinical laboratory analysis).

### 2.4. Statistical Analysis

Statistical analyses of the study were performed using SPSS, version 19.0 (SPSS Inc., United States) and MedCalc, version 12.3 (MedCalc Software Bvba, Ostend, Belgium).

Continuous variables were presented as median (interquartile range, 25th–75th percentile) for abnormal distribution data or mean ± standard deviation for normal distribution data. Categorical variables were expressed as number (percentage). Comparisons between groups were assessed using the Student *t*-test or the Mann-Whitney test for continuous variables or the *χ*^2^ or Fisher test for categorical variables when appropriate. The correlation of two-factor analysis was assessed with Spearman's rank correlation coefficient.

In order to determine factors associated with the abnormally elevated serum AFP level (>20 ng/mL) among patients with hepatitis B without developing HCC, univariable and multivariable logistic analyses were conducted. Only the factors significantly associated with the elevated serum AFP level in patients without HCC (*P* < 0.05) in the univariable analysis could enter into the multivariable logistic analysis. The multivariable logistic was processed using the forward stepwise regression method. The estimated odds ratio (OR) with its 95% confidence interval (CI) was presented in the study results.

The diagnostic performances of serum AFP and the AFP/(ALT × AST) ratio for the detection of HCC were evaluated using the area under the receiver operating characteristic (ROC) curves (AUCs). Comparisons of AUCs of different parameters for the diagnosis of HCC were assessed with DeLong tests. The optimal cutoff value is determined by the best Youden's index, which is calculated according to the following formula: Youden′s index = sensitivity + specificity–1. The sensitivity, specificity, positive likelihood ratio, negative likelihood ratio, and their 95% CIs were shown in the table.

For all analyses, all statistical tests were two-sided and *P* value < 0.05 was regarded as statistically significant.

## 3. Results

### 3.1. Clinical Characteristics

**Figure 1:**
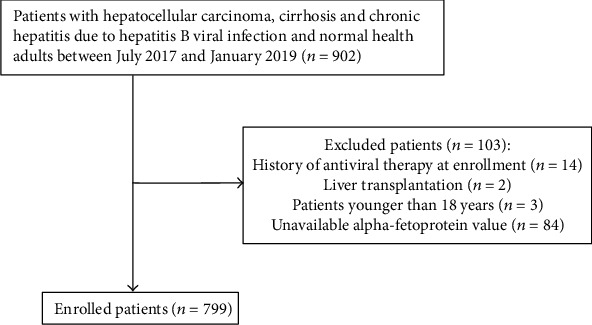
Flow chart of the study population.

The baseline characteristics of the enrolled patients are presented in Table 1. Of 902 patients recruited at the initial stage of the study, 799 patients were eligible for the study, including 240 patients with HCC (HCC group), 153 patients with cirrhosis (cirrhosis group), 248 patients with chronic hepatitis B (chronic hepatitis group), and 158 healthy adults (healthy control group). Of the enrolled patients, there were 637 male patients (79.7%, 637/799) and the median age was 46.0 years old (interquartile range: 36.0-56.0 years old). *P* values for comparisons between different groups are presented in Table 1. The AFP level and AFP/(ALT × AST) ratio in the HCC group were significantly higher than those in other groups (all *P* values < 0.001). The flow chart of the study population is shown in Figure 1.

### 3.2. Factors Associated with Elevated Serum AFP Levels in Patients with Cirrhosis and Chronic Hepatitis B

Table 2 shows the clinical and biochemical parameters correlated with the elevated serum AFP level (>20 ng/mL) in patients with cirrhosis and chronic hepatitis B. Univariable analysis showed that the male gender (*P* = 0.01), ALT level (*P* < 0.001), AST level (*P* < 0.001), alkaline phosphatase level (*P* = 0.045), gamma-glutamyl transferase level (*P* < 0.001), and prothrombin time (*P* = 0.02) were significantly associated with the elevated serum AFP level (>20 ng/mL) in patients with hepatitis B without developing HCC. In multivariable analysis, only the serum ALT level (*P* < 0.001) and gamma-glutamyl transferase level (*P* = 0.02) were significantly associated with the abnormally elevated serum AFP level in patients with hepatitis B. The increased serum ALT level was significantly correlated with the abnormally elevated AFP level (Rs = 0.395, *P* < 0.001) using Spearman's rank correlation coefficient.

### 3.3. Serum AFP Level in Patients with Cirrhosis and Chronic Hepatitis B with Different Serum ALT Levels

Table 3 presents serum AFP levels in the cirrhosis and chronic hepatitis B groups with different serum ALT levels. The median value of the serum AFP level was 4.0 ng/mL, 4.2 ng/mL, 38.6 ng/mL, and 43.2 ng/mL in the cirrhosis group with a normal ALT level, 1 upper limit of normal (ULN) < ALT level ≤ 2 ULN, 2 ULN < ALT level ≤ 5 ULN, and ALT level > 5 ULN, respectively. The median value of the serum AFP level in the cirrhosis group with a serum ALT level > 2 ULN was significantly higher than those with a serum ALT level ≤ 2 ULN (*P* < 0.001) whereas there was no significant difference between the median values of AFP levels in the cirrhosis group with a normal serum ALT level and those with 1 ULN < serum ALT level ≤ 2 ULN (*P* = 0.73).

For the chronic hepatitis B group, the median value of the serum AFP level was 2.1 ng/mL, 3.2 ng/mL, 6.6 ng/mL, and 42.7 ng/mL in the chronic hepatitis group with a normal ALT level, 1 upper limit of normal (ULN) < ALT level ≤ 2 ULN, 2 ULN < ALT level ≤ 5 ULN, and ALT level > 5 ULN, respectively. Similarly, the median value of the serum AFP level in the chronic hepatitis B group with a serum ALT level > 2 ULN was significantly higher than those with a serum ALT level ≤ 2 ULN (*P* < 0.001) whereas there was no significant difference between the median values of AFP levels in the chronic hepatitis B group with a normal serum ALT level and those with 1 ULN < serum ALT level ≤ 2 ULN (*P* = 0.05).

### 3.4. Performance of Serum AFP and the AFP/(ALT × AST) Ratio for the Diagnosis of HCC in Patients with Different Serum ALT Levels

#### 3.4.1. Comparison of Accuracy of AFP and the AFP/(ALT × AST) Ratio for Detecting HCC

The performance of AFP and the AFP/(ALT × AST) ratio for the diagnosis of HCC in patients with different ALT levels is shown in Table 4.

**Figure 2:**
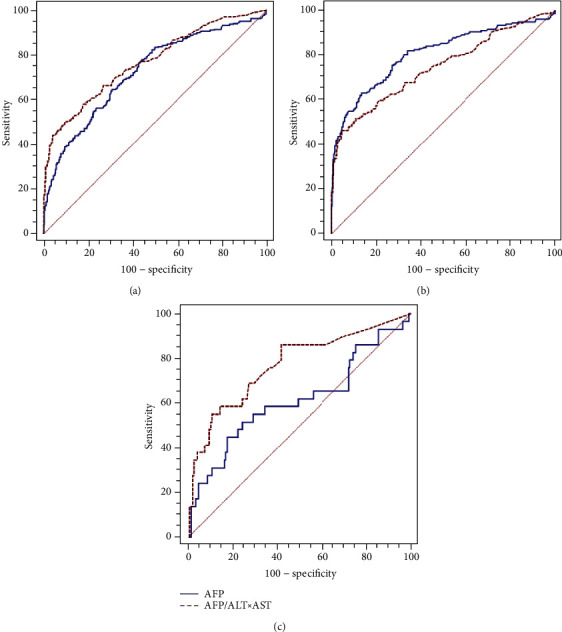
The area under the receiver operating characteristic curves (AUCs) of alpha-fetoprotein (AFP) and the AFP/[alanine transaminase (ALT) × aspartate aminotransferase (AST)] ratio for detecting hepatocellular carcinoma in the total cohort (a), patients with an ALT level ≤ 2 upper limit of normal (ULN) (b), and patients with an ALT level > 2 ULN (c).

As is shown in Figure 2, in the total population (*n* = 799), the performance of AFP (AUC: 0.725; 95% CI 0.691-0.756) was significantly lower than that of AFP/(ALT × AST) (AUC: 0.769; 95% CI 0.737-0.799) in the diagnosis of HCC (*P* < 0.001; Figure 2(a)). In patients with a serum ALT level ≤ 2 ULN, AFP showed better diagnostic accuracy compared to the AFP/(ALT × AST) ratio (*P* < 0.001; Figure 2(b)). In contrast, in patients with an ALT level > 2 ULN, the diagnostic performance of the AFP/(ALT × AST) ratio is significantly higher than that of AFP for HCC (*P* < 0.001; Figure 2(c)).

#### 3.4.2. Comparison of Accuracy of Parameters in Different Serum ALT Levels

Comparisons of AUCs showed that the AUC of serum AFP decreased significantly from 0.806 (95% CI 0.771-0.837) in patients with a serum ALT level ≤ 2 ULN to 0.611 (95% CI 0.535-0.683) in patients with a serum ALT level > 2 ULN (*P* < 0.001). Nevertheless, there was no significant difference between the AUCs of the AFP/(ALT × AST) ratio in patients with different ALT levels (0.745 vs. 0.769, respectively; *P* = 0.68).

## 4. Discussion

A high level of the serum AFP level is highly suspicious of hepatocellular carcinoma. However, the abnormally elevated serum AFP level was also found in patients with chronic hepatitis B or cirrhosis without evidence of HCC [6, 11, 20]. The present study illustrated that the serum ALT level has an independent effect on the serum AFP level and the elevated ALT level might impair the diagnostic performance of AFP tests for the detection of HCC. Our study found that the diagnostic performance of AFP tests was superior in patients with an ALT level ≤ 2 ULN (AUC 0.806; 95% CI 0.771-0.837) than in patients with an ALT level > 2 ULN (AUC 0.611; 95% CI 0.535-0.683; *P* < 0.001). In patients with an ALT level > 2 ULN, the AFP/(ALT × AST) ratio (AUC 0.769; 95% CI 0.700-0.829) was better than the AFP tumor (*P* < 0.001) marker in diagnosing HCC, with its sensitivity and specificity significantly increasing.

The main findings of the current study are in agreement with previous studies [15, 21]. The prevalence of abnormally elevated AFP levels in patients with chronic hepatitis C is about 10% to 43% in Western countries [22–24]. Previous studies have confirmed that the serum AFP level decreased or increased in parallel with the serum ALT level in noncancerous liver diseases [15–18]. Abnormally elevated AFP levels were found to decrease in response to antiviral therapy [12], suggesting that hepatic inflammation and viral replication were potential confounding factors for AFP tests in detection for HCC. However, to date, the underlying mechanism of the association between AFP levels and hepatic inflammation is still poorly understood, which needs to be further investigated in future studies. Our findings suggest that the measurement of AFP is not sufficient to detect HCC in patients with an ALT level > 2 ULN and dynamic observation of the serum AFP level during antiviral treatment is essential to avoid misleading results of AFP tests for diagnosing HCC.

In the present study, we also compared the diagnostic value of the AFP marker and AFP/(ALT × AST) ratio in diagnosing HCC. Liu et al. first used this novel index AFP/(ALT × AST) ratio in their study and found that the diagnostic value of the AFP/(ALT × AST) ratio was significantly better than that of AFP, with AUC up to 0.853 (95% CI 0.818–0.887) and 0.825 (95% CI 0.782–0.868) for differentiating HCC from non-HCC patients and from cirrhosis patients, respectively [19]. In the total cohort of our study, our data showed that the sensitivity of the AFP/(ALT × AST) ratio is not superior to that of AFP alone in the prediction of HCC with its AUC slightly improved. However, among patients with ALT > 2 ULN, the performance of the AFP/(ALT × AST) ratio performed significantly better than that of AFP alone for detecting HCC. That being said, the AFP/(ALT × AST) ratio was more recommended than that of AFP alone targeted on patients with significantly elevated ALT levels (ALT > 2 ULN). Notably, our data also found that there was no significant difference in the diagnostic performance of the AFP/(ALT × AST) ratio in patients with different ALT levels, suggesting that the AFP/(ALT × AST) ratio could avoid the influences of hepatic inflammatory factors in detecting HCC. However, the diagnostic efficacy of this new index for diagnosing HCC in patients with various liver diseases needs to be validated in future studies.

Unique features of our current study are the focus on the impact of serum ALT levels on the diagnostic performance of AFP tests for the detection of HCC. The study specifically analyzed the value of the serum AFP level in patients with chronic hepatitis B and cirrhosis according to different ALT levels, illustrating ALT > 2 ULN might significantly affect AFP. On the other hand, our data strongly suggest that AFP is a good test for the detection of HCC in patients with hepatitis B with an ALT level ≤ 2 ULN. And the index AFP/(ALT × AST) ratio was better than AFP for detection of HCC in patients with ALT > 2 ULN.

There were several limitations to our study. First, this was a retrospective single-center study. The current findings of our study need to be validated in a future large, prospective multicenter study. Second, there are many different cofactors that might affect ALT levels. Due to the retrospective design of our study, we failed to control and discuss related cofactors of serum ALT in our analysis. However, as chronic hepatitis B infection is still a major health problem in China and significant serum ALT elevation was often observed in patients with chronic hepatitis B, there is a great need for assessing the influence of ALT levels on the diagnostic accuracy of the noninvasive method for the detection of HCC in the population. The data presented in this study provide further evidence that the serum ALT level is a very important factor in the accuracy of AFP for predicting HCC. Future studies that control and discuss related cofactors of serum ALT on the performance of AFP in detecting HCC are thus needed. Third, our study excluded patients with antiviral treatments at study entry and therefore failed to further analyze the impact of ALT levels on AFP for predicting HCC among patients with antiviral treatments. However, antiviral treatment might be a cofactor that may affect our study results. Future studies are needed to better clarify whether there is an impact of antiviral treatments on serum ALT when AFP was used as a predictor for HCC. Finally, our study population is limited to hepatitis viral B infection. The results of the current study might not be applied to other liver diseases.

## 5. Conclusions

In conclusion, our study proposed that the elevated serum ALT level is strongly associated with the abnormally elevated AFP level in patients with hepatitis B without developing HCC. The diagnostic value of AFP tests might be increased by adjusting for serum ALT values with better predictive values in patients with an ALT level ≤ 2 ULN and impaired predictive performance in patients with an ALT level > 2 ULN. The AFP/(ALT × AST) ratio had a better diagnostic performance than AFP in patients with elevated ALT levels.

## Figures and Tables

**Table 1:** Baseline demographic data of the study population (*n* = 799).

Variable	HCC (*n* = 240)	Cirrhosis (*n* = 153)	Chronic hepatitis (*n* = 248)	Healthy group (*n* = 158)	*P*_1_ value	*P*_2_ value	*P*_3_ value
Demographic							
Age (years)	53.0 (44.0-62.0)	53.0 (47.0-62.0)	42.0 (34.0-49.0)	34.0 (28.0-45.0)	0.26	<0.001	<0.001
Sex, male	221 (92.1%)	110 (71.9%)	197 (79.4%)	109 (69.0%)	<0.001	<0.001	<0.001
Hematological data							
HGB (g/L)	139.0 (127.0-151.0)	117.0 (97.0-134.0)	142.0 (128.0-153.5)	151.0 (136.0-158.0)	<0.001	0.24	<0.001
PLT (10^9^/L)	169.9 (120.0-224.0)	89.0 (63.5-126.5)	180.0 (139.0-229.0)	251.0 (217.0-285.5)	<0.001	0.08	<0.001
PT (s)	13.8 (13.3-14.5)	16.2 (14.7-18.5)	14.4 (13.3-16.9)	12.9 (12.4-13.3)	<0.001	<0.001	<0.001
Liver function tests							
AST (IU/L)	32.0 (25.0-48.8)	49.0 (32.5-74.0)	47.5 (27.0-213.5)	19.0 (16.0-22.0)	<0.001	<0.001	<0.001
ALT (IU/L)	34.5 (24.0-49.8)	35.0 (23.0-56.5)	65.5 (27.0-413.8)	19.0 (13.8-27.0)	0.43	<0.001	<0.001
GGT (IU/L)	55.5 (32.4-98.0)	53.0 (35.0-136.0)	47.0 (22.0-161.0)	22.0 (16.0-34.0)	0.42	0.20	<0.001
ALP (IU/L)	85.0 (67.0-117.3)	104.0 (81.0-147.0)	86.5 (67.0-123.3)	60.0 (48.0-68.0)	<0.001	0.88	<0.001
Tumor markers							
AFP (ng/mL)	18.6 (4.8-200.0)	5.1 (2.7-12.1)	4.6 (1.9-36.5)	2.8 (2.0-3.9)	<0.001	<0.001	<0.001
AFP/(ALT × AST) (10^−3^)^#^	17.6 (4.3-122.0)	3.3 (1.3-8.2)	1.7 (0.3-4.6)	8.6 (3.7-14.1)	<0.001	<0.001	<0.001

Data were presented as median (interquartile range, 25th–75th percentile) or number (percentage). HCC: hepatocellular carcinoma; HGB: hemoglobin; PLT: platelet count; PT: prothrombin time; AFP: alpha-fetoprotein; ALT: alanine aminotransferase; AST: aspartate aminotransferase; ALP: alkaline phosphatase; GGT: gamma-glutamyl transferase. ^#^For the convenience of representation, the value of AFP/(ALT × AST) was 1000 times the actual value. *P*_1_ value was given for the comparison of the HCC and cirrhosis groups; *P*_2_ value was given for the comparison of the HCC and chronic hepatitis groups; *P*_3_ value was given for the comparison of the HCC and healthy control groups.

**Table 2:** Univariable and multivariable logistic regression analyses of factors associated with elevated AFP (≥20 ng/mL) in a cohort with chronic hepatitis B and cirrhosis (*n* = 401).

Variable	Univariable		Multivariable	
	OR (95% CI)	*P* value	OR (95% CI)	*P* value
Age (years)	1.00 (0.98, 1.02)	0.95		
Sex, male	2.52 (1.25, 5.11)	0.01		
AST (IU/L)	1.01 (1.00, 1.01)	<0.001		
ALT (IU/L)	1.01 (1.00, 1.01)	<0.001	1.00 (1.00, 1.01)	<0.001
GGT (IU/L)	1.01 (1.00, 1.01)	<0.001	1.00 (1.00, 1.00)	0.02
ALP (IU/L)	1.01 (1.00, 1.01)	0.045		
HGB (g/L)	1.00 (1.00, 1.01)	0.43		
PLT (10^9^/L)	1.00 (1.00, 1.00)	0.09		
PT (s)	1.08 (1.02, 1.14)	0.02		

AFP: alpha-fetoprotein; OR: odds ratio; CI: confidence interval; ALT: alanine aminotransferase; AST: aspartate aminotransferase; ALP: alkaline phosphatase; GGT: gamma-glutamyl transferase; HGB: hemoglobin; PLT: platelet count; PT: prothrombin time.

**Table 3:** Values of serum AFP (ng/mL) levels in patients with chronic hepatitis B and cirrhosis (*n* = 401) with different alanine aminotransferase (ALT) levels.

ALT level (IU/L)	Cirrhosis (*n* = 153)	Chronic hepatitis (*n* = 248)
Normal	4.0 (2.6-8.2) (*n* = 78)	2.1 (1.5-3.5) (*n* = 86)
1–2 ULN	4.2 (2.1-7.5) (*n* = 48)	3.2 (1.6-7.2) (*n* = 44)
2-5 ULN	38.6 (8.5-94.5) (*n* = 19)	6.6 (2.3-80.9) (*n* = 36)
>5 ULN	43.2 (20.5-149.1) (*n* = 8)	42.7 (13.7-113.9) (*n* = 82)

Data were presented as median (interquartile range, 25th–75th percentile). AFP: alpha-fetoprotein; ALT: alanine aminotransferase; ULN: upper limit of normal.

**Table 4:** Performance of AFP and the AFP/(ALT × AST) ratio for the diagnosis of HCC in patients with different ALT levels.

Parameter	Cutoff	AUC (95% CI)	*P* value^#^	Sensitivity	Specificity	LR+	LR-
AFP (ng/mL)			<0.001				
Total cohort	7.8	0.725 (0.691-0.756)		0.65 (0.58-0.71)	0.72 (0.68-0.75)	2.27 (2.0-2.5)	0.49 (0.4-0.6)
Cohort with ALT ≤ 2 ULN	7.8	0.806 (0.771-0.837)		0.63 (0.56-0.70)	0.88 (0.84-0.91)	5.07 (4.5-5.7)	0.42 (0.3-0.6)
Cohort with ALT > 2 ULN	88.3	0.611 (0.535-0.683)		0.52 (0.33-0.71)	0.76 (0.68-0.83)	2.15 (1.5-3.1)	0.64 (0.4-1.0)
AFP/(ALT × AST) (10^−3^)^&^			0.68				
Total cohort	18.0	0.769 (0.737-0.799)		0.50 (0.44-0.57)	0.91 (0.88-0.93)	5.32 (4.7-6.1)	0.55 (0.4-0.7)
Cohort with ALT ≤ 2 ULN	28.3	0.745 (0.707-0.780)		0.46 (0.40-0.53)	0.95 (0.93-0.97)	9.92 (8.6-11.5)	0.56 (0.3-0.9)
Cohort with ALT > 2 ULN	5.6	0.769 (0.700-0.829)		0.59 (0.39-0.77)	0.86 (0.79-0.91)	4.13 (3.0-5.6)	0.48 (0.3-0.9)

AFP: alpha-fetoprotein; ALT: alanine aminotransferase; AST: aspartate aminotransferase; HCC: hepatocellular carcinoma; AUC: area under the receiver operating characteristic curve; CI: confidence interval; LR+: positive likelihood ratio; LR-: negative likelihood ratio; ULN: upper limit of normal. ^&^For the convenience of representation, the value of AFP/(ALT × AST) was 1000 times the actual value. ^#^*P* value was calculated for comparison of AUCs in patients with ALT ≤ 2 ULN and those with ALT > 2 ULN.

## Data Availability

The raw/processed data required to reproduce these findings cannot be shared at this time as some of the data also forms part of an ongoing study.

## Abbreviations

HCC: Hepatocellular carcinoma
ALT: Alanine transaminase
AFP: Alpha-fetoprotein
AST: Aspartate aminotransferase
AUC: Area under the receiver operating characteristic curve.

## Additional Points

*Highlights*. Serum ALT was significantly associated with elevated AFP in non-HCC cases. Higher ALT levels might impair the accuracy of AFP for diagnosing HCC. AFP/(ALT × AST) was better than AFP in patients with elevated ALT for detecting HCC.
